# Impact of lauric acid supplementation on bovine oocyte maturation, IVF embryo development, and stress protection during IVM

**DOI:** 10.1590/1984-3143-AR2025-0095

**Published:** 2026-05-01

**Authors:** Nguyen Duc Truong, Do Thi Kim Lanh, Nguyen Thi Ngoc Anh, Nguyen Hoai Nam, Bui Van Dung, Nguyen Van Thanh

**Affiliations:** 1 Faculty of Veterinary Medicine, Vietnam National University of Agriculture, Hanoi, Vietnam

**Keywords:** lauric acid, bovine oocytes, *in vitro* maturation, heat stress, oxidative stress

## Abstract

This study investigated the effects of lauric acid (LA) supplementation during *in vitro* maturation (IVM) of bovine oocytes on nuclear maturation and subsequent embryonic development. Cumulus–oocyte complexes (COCs) were matured in medium containing LA at concentrations of 50, 100, 150, 200, or 300 µM, or without LA (control). Embryo development following *in vitro* fertilization (IVF) and culture (IVC) was assessed. Based on optimal outcomes in the first experiment (A total of 1,558 oocytes were used across 6 - 8 replicates per treatment group),200 µM LA was selected to evaluate its protective role under cellular stress. Oocytes (n = 1,255; five replicates) were matured with or without 200 µM LA and exposed to heat stress (41 °C) or oxidative stress (1 mM H_2_O_2_). LA supplementation significantly improved blastocyst rates, with 100 - 200 µM groups showing higher formation rates (≥ 37.1%) compared to control (21.3%) (p < 0.05). Blastocyst quality, based on A–B grade embryos, was also enhanced in the 200 µM (51.1%) and 300 µM (56.5%) groups versus control (19.2%) and 50 µM (16.9%) (p < 0.05). Stress exposure reduced maturation and blastocyst rates. Oxidative stress significantly decreased maturation (OS: 44.9%) and blastocyst development (18.6%) compared to control (72.3% and 28.4%, respectively), while LA treatment improved these outcomes (OS-LA: 51.5% and 31.5%) (p < 0.05). Under heat stress, LA showed a non-significant trend toward higher blastocyst rates (24.8%) compared to stress alone (16.2%). Under normal conditions, 200 µM LA significantly enhanced blastocyst yield (44.2%) versus control (28.4%) (p < 0.05). Data were analyzed using ANOVA with post-hoc tests, and differences were considered significant at p < 0.05. These results indicate that LA supplementation during IVM enhances bovine embryo developmental competence and partially mitigates oxidative stress-induced impairment.

## Introduction

*In vitro* maturation (IVM) of oocytes is a critical process in bovine reproductive biotechnology, particularly for assisted reproductive technologies (ART) such as *in vitro* fertilization (IVF), which are widely used in both research and commercial livestock industries (Lonergan and Fair, 2015). The ability to produce high-quality embryos through IVF has revolutionized bovine genetics, enabling efficient breeding programs, genetic improvement, and preservation of valuable cattle lines. Furthermore, IVM and IVF facilitate the production of embryos for cloning, transgenic research, and conservation of endangered species. However, the success of bovine embryo production is influenced by multiple factors that can impair oocyte maturation and, subsequently, the developmental competence of the embryos. Key challenges include oxidative stress, heat stress, and suboptimal culture conditions, which can hinder oocyte maturation and lower fertilization rates. These stresses are especially problematic in the context of commercial livestock production, where environmental factors and reproductive efficiency are of paramount importance. As a result, optimizing IVM conditions and improving embryo quality remain significant challenges in both research and applied bovine reproductive biotechnology (Ferre et al., 2020; Feng et al., 2024).

Heat stress is a common challenge in modern livestock management, particularly under conditions of climate change, and has been shown to impair oocyte maturation and the subsequent fertilization process. The detrimental effects of heat stress on oocyte quality and embryo development are well-documented and include reduced maturation rates, decreased fertilization efficiency, and impaired embryo quality, which can lower reproductive success (Mietkiewska et al., 2022; Joyce et al., 2024). Moreover, oxidative stress, characterized by an imbalance between the production of reactive oxygen species (ROS) and the body’s antioxidant defense mechanisms, is a major factor that contributes to oocyte damage and reduced developmental competence (Behrman et al., 2001) as well as in the fertilization capacity of bovine spermatozoa and in the interaction between spermatozoa and oocytes (Goncalves et al., 2010). Both oxidative and heat stress are known to negatively impact cellular structures, including lipids, proteins, and DNA, leading to compromised oocyte maturation and embryo viability (Soto-Heras and Paramio, 2020).

To counteract these detrimental effects, various fatty acids and antioxidants have been investigated in assisted reproductive technologies (ART). For example, linoleic acid and palmitic acid modulate oocyte maturation and energy metabolism (Marei et al., 2010; Aardema et al., 2011), while melatonin has been widely applied as an antioxidant to reduce ROS and improve porcine IVM outcomes (Do et al., 2015a; Islam et al., 2025). Importantly, our research group has also investigated chlorogenic acid (CGA), a polyphenolic antioxidant, and demonstrated its beneficial effects in protecting porcine oocytes against oxidative and heat stress as well as improving embryo development (Nguyen et al., 2017, 2019, 2023). These studies provide a comparative foundation supporting the rationale for evaluating lauric acid (LA) supplementation during bovine IVM.

Lauric acid, a medium-chain fatty acid (MCFA) found in various dietary lipids, has attracted attention for its antioxidant, anti-inflammatory, and metabolic regulatory properties (Namachivayam and Valsala Gopalakrishnan, 2023). Although studies of LA in reproductive biology are limited, its ability to promote mitochondrial function, regulate lipid metabolism, and strengthen antioxidant defenses suggests that it may have potential to improve oocyte maturation and embryo development, particularly under stress conditions. Given these properties, lauric acid may enhance oocyte quality by mitigating oxidative stress and improving the microenvironment for maturation and fertilization when included during IVM. Building on this rationale, the present study investigates the effects of lauric acid on bovine oocyte maturation, *in vitro* fertilization (IVF) embryo developmental competence, and its protective effects under heat and oxidative stress. By addressing this gap, we aim to provide preliminary evidence for the potential application of LA as an additive to improve bovine ART efficiency under environmentally challenging conditions.

## Methods

As no live animals were used in this study, no ethical approval was required at any of the participating institutions.

### Oocyte collection, *in vitro* maturation, fertilisation, and embryo culture

Ovaries were collected from slaughtered cows at abattoirs in Thuong Tin and Phu Xuyen, Hanoi, Vietnam. The ovaries were transported to the laboratory in physiological saline solution within 1 hour at 30°C. Ovaries were washed three times with phosphate-buffered saline (m-PBS; Sigma-Aldrich, USA) supplemented with 100 IU/mL penicillin G potassium (Sigma, USA) and 0.1 mg/mL streptomycin sulfate (Sigma, USA). Cumulus–oocyte complexes (COCs) were aspirated from follicles measuring 2 -6 mm in diameter using Medium 199 with Hank's salts (Gibco, USA) supplemented with 20 mM HEPES. Only COCs with uniformly dark cytoplasm and intact cumulus cell masses were selected for experiments.

Approximately 50 COCs were matured in 500 µL of maturation medium consisted of TCM199 with Earle’s salts (Invitrogen, Carlsbad, CA, USA) supplemented with 0.6 mM cysteine (Sigma-Aldrich, St. Louis, MO, USA), 0.02 AU/mL follicle stimulating hormone (FSH-Kyoritsuseiyaku Co., Tokyo, Japan), 5% foetal bovine serum (FBS; Invitrogen), and 50 µg/mL gentamicin (Sigma-Aldrich). Oocytes were incubated in 4-well dishes (SPL life science, Korea) for 22 hours at 38.5 °C in a humidified incubator (MEMMERT ICO105med, Germany) containing 5% CO_2_ in air. *In vitro* fertilisation (IVF) was performed according to the procedures described by Isobe et al. (2015) with minor modifications. After maturation, the COCs were fertilised *in vitro* with frozen–thawed spermatozoa (5 × 10^6^ cells/mL) from a single bull (Vietnamese H’mong) in fertilisation medium (IVF100; Research Institute for the Functional Peptides Co., Yamagata, Japan). Briefly, cryopreserved semen was thawed at 37 °C and washed twice with the fertilisation medium via centrifugation at 630 × g for 5 min. The sperm pellet was resuspended in the fertilisation medium to achieve a concentration of 4 × 10^6^ cells/mL. The spermatozoa (250 µL) were added to 250 µL of fertilisation medium containing 50 matured oocytes in 4-well dishes. The final sperm concentration was adjusted to 2 × 10^6^ cells/mL, as recommended in standard bovine IVF protocols (Parrish, 2014; Gordon, 2003). The oocytes were co-incubated with spermatozoa for 6 h at 38.5 °C in a humidified incubator containing 5% CO_2_ in air.

After insemination, putative zygotes were denuded of cumulus cells and adherent sperm via mechanical pipetting. The zygotes were cultured in a modified synthetic oviduct fluid (mSOF) medium supplemented with 4 mg/ml bovine serum albumin (BSA; SigmaAldrich) and 50 μg/ml of gentamicin for 72 hr at 38 °C in a humidified atmosphere of 5% CO_2_ and 5% O_2_. After 72 hr of culture, only cleaved embryos were further cultured in mSOF supplemented with 5% FBS at 38 °C in a humidified 5% CO_2_ atmosphere for an additional 5 days to evaluate their ability to develop to the blastocyst stage.

### Experimental design

**Effect of LA Supplementation:** To evaluate the effect of LA supplementation in the maturation medium on oocyte maturation rate and embryo developmental competence, oocytes were cultured in maturation medium supplemented with LA (Sigma - Aldrich) at different concentrations of 50, 100, 150, 200, 300 µM, or without LA supplementation (control group). The LA concentrations were determined based on preliminary tests and reference to studies using LA *in vitro*. LA stock solution (1 mM) was first prepared by dissolving lauric acid in dimethyl sulfoxide (DMSO). The stock solution was then diluted with the IVM medium to obtain a final concentration of 300 µM, and serial dilutions were prepared from this medium to achieve final concentrations of 200, 150, 100, and 50 µM. The control group received an equivalent volume of DMSO to ensure that solvent exposure was consistent across all treatments. Oocyte maturation rate (MII %), cleavage rate, blastocyst rate, and total cell number/blastocyst were evaluated to determine the optimal LA concentration in the oocyte maturation medium. Each treatment group included a total of approximately 200 – 280 oocytes, and the experiments were conducted in six to eight independent replicates.
**Heat Stress Protection:** To evaluate the protective capacity of lauric acid against heat stress, oocytes were matured in TCM199 medium with LA supplementation (at the optimal concentration determined from the first experiment) or without LA (control group). The maturation process was conducted in a humidified incubator with 5% CO_2_ at either 38.5 °C or 41 °C (heat stress group). After IVM, the oocytes proceeded through IVF and IVC as described above.
**Oxidative Stress Protection:** To evaluate the protective capacity of lauric acid against oxidative stress, oocytes were matured in TCM199 medium with LA supplementation (at the optimal concentration) or without LA (control group). The maturation process was conducted in a humidified incubator with 5% CO2 at 38.5 °C. Based on previous research showing that 1 mM H_2_O_2_ induces apoptosis in porcine oocytes (Do et al., 2015b), oocytes were matured in IVM medium with or without optimal LA supplementation under the influence of 1 mM H_2_O_2_. After IVM, the oocytes proceeded through IVF and IVC.

For the stress experiments, a total of approximately 1,255 oocytes were used across five independent replicates (n = 5).

### Assessment methods

**Figure 1:**
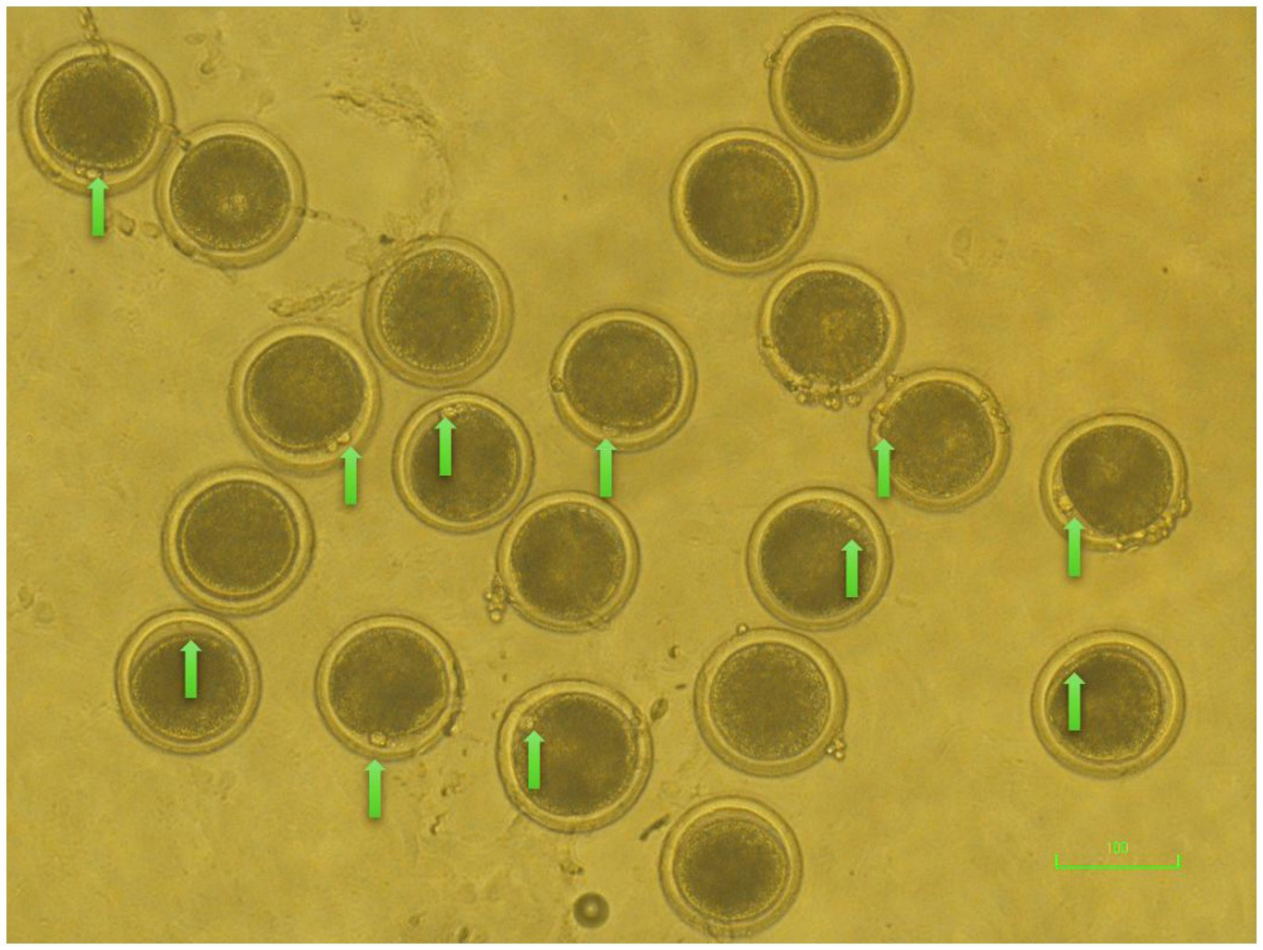
Representative image of bovine oocytes showing extrusion of the first polar body (green arrows), confirming nuclear maturation to the metaphase II (MII) stage. This image is provided as a representative example only and is not intended as quantitative evidence. Scale bar = 100 µm.

After *in vitro* maturation (IVM) for 22 h, cumulus–oocyte complexes (COCs) were subjected to *in vitro* fertilization (IVF) as described above. After 6 h of insemination, the oocytes were denuded by gentle pipetting to remove cumulus cells and attached spermatozoa. The denuded oocytes were then examined under a stereomicroscope. Oocytes exhibiting extrusion of the first polar body (PB1, Figure 1) were considered to have reached the metaphase II (MII) stage, as previously described for bovine oocyte maturation assessment (van der Westerlaken et al., 1994; Park et al., 2005; Aguila et al., 2020). The proportion of oocytes showing PB1 extrusion was recorded as the MII rate (%).

**Figure 2:**
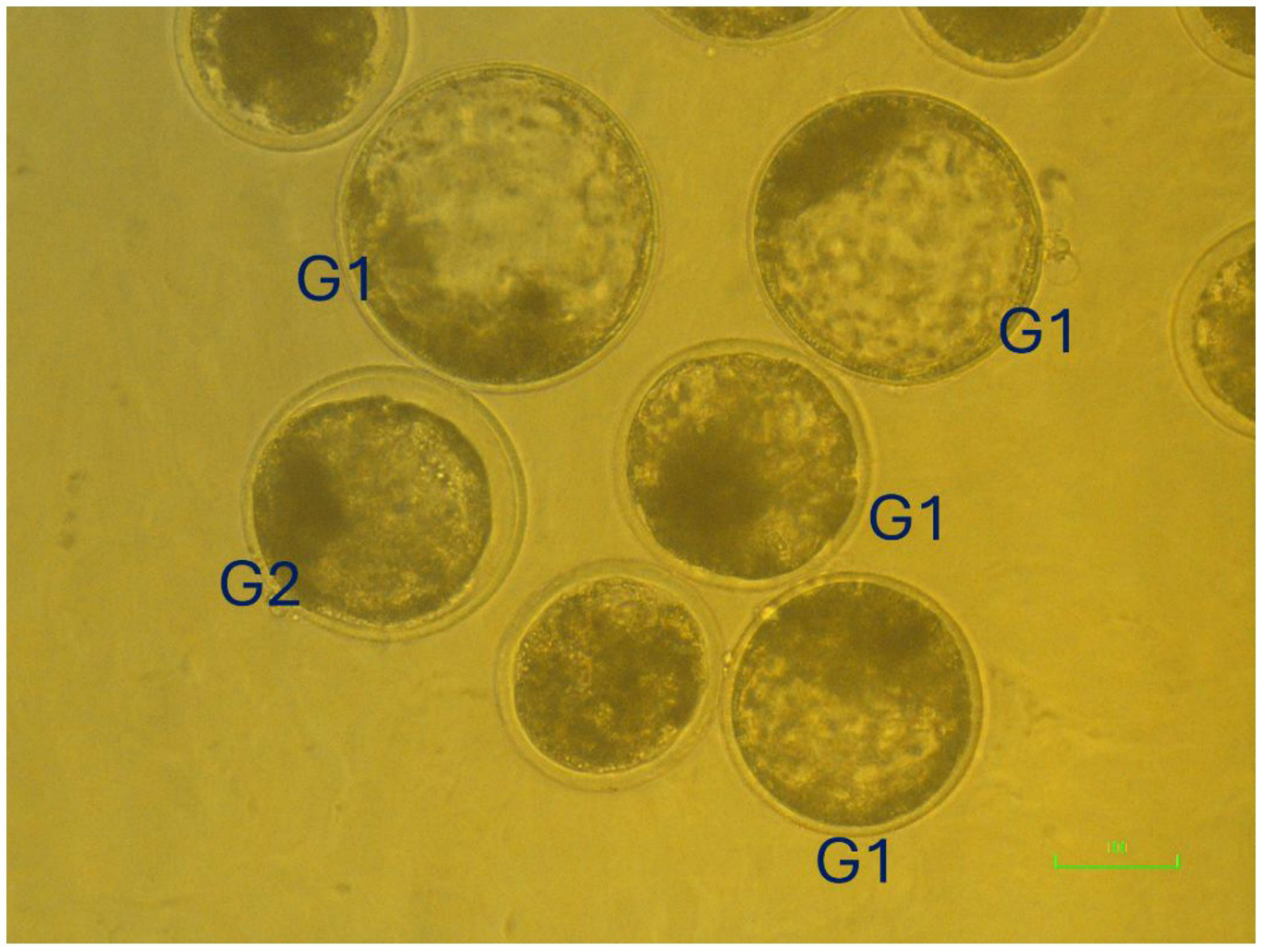
Representative image of day 7 *in vitro* produced bovine blastocysts classified according to the International Embryo Technology Society (IETS) standards (Stringfellow and Givens, 2010). Grade 1 (G1, Excellent/Good): embryos are symmetrical and spherical in shape, with uniform cell size, color, and density. At least 85% of the embryonic mass is intact. Grade 2 (G2, Fair): embryos exhibit moderate irregularities in shape, size, or color of blastomeres, but at least 50% of the embryonic mass remains intact. This figure shows representative examples only and is not intended as quantitative evidence. Scale bar = 100 µm.

Development to the four-cell embryo stage (cleaved) and blastocyst formation was evaluated after 24 h and 7 days of culture, respectively. Morphological grading of day 7 blastocysts was performed under an inverted microscope according to the International Embryo Technology Society (IETS) Manual, 4th Edition (Stringfellow and Givens, 2010). Embryos were classified into Grade 1 (excellent or good) and Grade 2 (fair) based on overall morphology, blastocoel expansion, cellular uniformity, and degree of fragmentation (Figure 2).

- Grade 1: Spherical, symmetrical blastocysts with uniform, evenly sized blastomeres and no visible fragmentation.
- Grade 2: Slightly irregular shape, minor variation in blastomere size, or small areas of fragmentation (< 20%).

### Statistical analyses

The rates of oocyte maturation (MII%), fertilization, cleavage and blastocyst formation were used as main outcome variables. Percentage data were subjected to arcsine square-root transformation to stabilize variance and analyzed by one-way ANOVA or two-way ANOVA (as applicable) using StatView (Abacus Concepts, Berkeley, CA, USA). When ANOVA showed significance, means were compared using Fisher’s protected least significant difference (PLSD) test. The proportion of mutated blastocysts was analyzed by chi-squared test with Yates’ correction.

Comparisons were made among LA concentration groups (Experiment 1), and among control, oxidative stress (OS), and heat stress (HS) conditions, each with or without LA supplementation (Experiment 2). All data are expressed as mean ± SEM, and *p* < 0.05 was considered significant.

## Results

### Effects of LA supplementation during IVM on maturation and developmental competence of bovine oocytes

To evaluate the effects of different concentrations of LA presented during IVM on the maturation rate and development of bovine IVF embryos, cumulus–oocyte complexes (COCs) were matured in medium supplemented with 0, 50, 100, 150, 200, and 300 µM LA. Following maturation, oocytes were subjected to *in vitro* fertilization (IVF) and subsequent *in vitro* culture (IVC).

As listed in Table 1, supplementation of the maturation medium with various concentrations of LA did not significantly influence the maturation rate of bovine oocytes compared to the control group (0 µM LA, p > 0.05). The maturation rates ranged from 77.01% for the control to a high of 86.57% observed at 150 µM LA.

**Table 1:** Effect of lauric acid supplementation during IVM on the maturation of bovine oocytes.

Concentration of Lauric acid (µM)	No. of examined oocytes	No. of matured oocytes	Maturation rate (%)
0	261	201	77.01 ± 4.5
50	258	220	85.27 ± 4.2
100	283	227	80.64 ± 3.0
150	283	245	86.57 ± 3.4
200	256	213	83.20 ± 3.7
300	217	185	85.25 ± 3.1

Six to eight Data are expressed as the mean ± SEM from six to eight replications, comprising approximately 200 - 280 oocytes per treatment.

Similarly, the cleavage rates of oocytes matured with different LA concentrations did not show significant differences among the groups (p > 0.05). Table 2 shows cleavage rates varying from 68.84% (300 µM LA) to 81.94% (50 µM LA).

**Table 2:** Effect of lauric acid supplementation during IVM on the cleavage rate and blastocyst formation following in vitro fertilization.

Concentration of Lauric acid (µM)	No. of examined oocytes	No. of cleaved oocytes	Cleavage rate (%)
0	221	160	72.40 ± 2.7
50	227	186	81.94 ± 3.1
100	241	184	76.0 ± 3.5
150	257	209	81.32 ± 2.3
200	248	183	73.79 ± 4.5
300	215	148	68.84 ± 5.1

Data are expressed as the mean ± SEM from six to eight replications, comprising approximately 220 - 260 oocytes per treatment.

However, supplementation with LA during IVM significantly impacted the developmental competence of the resulting IVF embryos, specifically the blastocyst formation rate and the quality of blastocysts. As presented in Table 3 , the blastocyst rate in the control group (21.27%) was significantly lower (p < 0.05) than that of the other groups supplemented with 100 µM (36.74%), 150 µM (38.52%), and 200 µM (37.10%) LA. Regarding blastocyst quality, assessed by the rate of grade 1 - 2 class embryos, the control group (19.15%) and the 50 µM LA group (16.87%) exhibited significantly lower rates (p < 0.05) compared to the 200 µM (51.09%) and 300 µM (56.45%) LA groups.

**Table 3:** Effect of lauric acid supplementation during IVM on developmental competence of IVF bovine oocytes.

Concentration of Lauric acid (µM)	No. of examined oocytes	No. of Blastocyst formation	Blastocyst rate (%)	No. of embryo (Grade I - II)	I – II grade Embryos rate (%)
0	221	47	21.27 ± 3.3b	9	19.15 ± 7.7^b^
50	227	83	36.56 ± 7.9^ab^	14	16.87 ± 10.5^b^
100	241	89	36.74 ± 5.2a	29	32.60 ± 12.0^ab^
150	257	99	38.52 ± 6.0^a^	25	25.25 ± 7.5^b^
200	248	92	37.10 ± 3.5^a^	47	51.09 ± 9.0^a^
300	215	62	28.84 ± 3.8^ab^	35	56.45 ± 10.5^a^

Data are expressed as the mean ± SEM from six to eight replications, comprising approximately 220 - 260 oocytes per treatment. Embryos were classified based on morphological criteria following IETS guidelines (2020); Grade 1 - 2 indicates good to fair quality blastocysts. ^a,b^Values with different superscripts in the same column are significantly different (p < 0.05).

### Effects of LA supplementation during IVM on maturation rate and developmental competence of oocytes exposed to stress

Based on the optimal concentration determined from the previous experiment, further studies were conducted using 200 µM LA to assess its protective effects under heat stress (41 °C) and oxidative stress (1 mM H_2_O_2_) conditions.

As shown in Table 4, maturation rates were significantly reduced (p < 0.05) in the oxidative stress groups (OS: 44.9 ± 1.7%; OS-LA: 51.5 ± 2.7%) compared to the control (Control: 72.3 ± 7.8%; Control-LA: 75.1 ± 5.3%) and heat stress groups (Heat: 67.5 ± 1.9%; Heat-LA: 72.7 ± 1.4%). However, LA supplementation (200 µM) did not significantly improve the maturation rate under either heat stress or oxidative stress conditions compared to the respective stress groups without LA supplementation.

**Table 4:** Effects of oxidative (1 mM H_2_O_2_) and heat stress (41 °C) during in vitro maturation (IVM) on oocyte maturation in the presence or absence of lauric acid (200 µM).

Treatment	No. of examined oocytes	No. of matured oocytes	Maturation rate (%±SE)
Control-LA	203	152	75.1 ± 5.3a
Control	203	146	72.3 ± 7.8^a^
Heat	200	135	67.5 ± 1.9^a^
Heat-LA	198	144	72.7 ± 1.4^a^
OS-LA	237	123	51.5 ± 2.7b
OS	214	92	44.9 ± 1.7^b^

Data are expressed as the mean ± SEM from five replication, comprising approximately 200 oocytes/ treatment. ^a,b^Values with different superscripts in the same column are significantly different (p < 0.05).

Evaluation of developmental competence under stress conditions revealed significant impacts on blastocyst formation (Table 5). Both heat stress (16.2 ± 2.6%) and oxidative stress (18.6 ± 4.2%) significantly reduced the blastocyst formation rate compared to the control group without LA (28.4 ± 4.0%). Under normal culture conditions, supplementation with 200 µM LA significantly increased the blastocyst rate (44.2 ± 3.8%) compared to the control without LA (28.4 ± 4.0%) (p < 0.05). Importantly, under oxidative stress, 200 µM LA supplementation significantly improved the blastocyst rate (31.5 ± 6,0) compared to the OS group without LA (18.6 ± 4.2%) (p < 0.05). This indicates a protective effect of LA against oxidative stress-induced developmental impairment. Under heat stress, the blastocyst rate with LA (24.8 ± 2.9%) was numerically higher than without LA (16.2 ± 2.6%), but this difference was not statistically significant based on the provided superscripts.

**Table 5:** Effects of oxidative (1 mM H_2_O_2_) and heat stress (41 °C) during in vitro maturation (IVM) on embryo development in the presence or absence of lauric acid (200 µM).

Treatment	No. of examined embryos	No. of cleaved embryos	Cleavage rate (%±SE)	No. of embryo develop to blastocyst stage	Blastocyst rate (%±SE)
Control-LA	166	137	82.3 ± 2.6^a^	74	44.2 ± 3.8a
Control	150	100	69.0 ± 8.0^ab^	42	28.4 ± 4.0b
Heat	191	119	62.6 ± 5.1^b^	31	16.2 ± 2.6^c^
Heat-LA	183	116	63.1 ± 3.1^b^	45	24.8 ± 2.9^bc^
OS-LA	105	77	74.2 ± 2.7^ab^	30	31.5 ± 6.0^b^
OS	111	70	63.9 ± 8.7^b^	19	18.6 ± 4.2^c^

Data are expressed as the mean ±SEM from five replications, comprising approximately 105 – 190 embryos/ treatment. ^a,b^Values with different superscripts in the same column are significantly different (p < 0.05).

Assessment of blastocyst quality by the hatching rate showed no statistically significant differences among any of the treatment groups under normal, heat stress, or oxidative stress conditions with or without 200 µM LA (Table 6).

**Table 6:** Effect of oxidative (1 mM H_2_O_2_) and heat stress (41 °C) during in vitro maturation (IVM) on blastocyst quality in the presence or absence of lauric acid (200 µM).

Treatment	No. of examined blastocyts	No. of hatched embryos	Hatching rate (%±SE)
Control-LA	74	26	35.7 ± 2.6
Control	42	10	28.8 ± 10.9
Heat	31	8	34.2 ± 16.5
Heat-LA	45	18	41.6 ± 8.0
OS-LA	30	12	43.2 ± 11.8
OS	19	3	18.3 ± 13.0

Five replicate trials were carried out. Data are expressed as the mean ±SEM.

## Discussion

The present study investigated the effects of Lauric Acid (LA) supplementation during *in vitro* maturation (IVM) on bovine oocyte maturation and embryonic development, with a focus on its potential to confer protection against heat and oxidative stress. Our findings indicate that LA during IVM supplementation improves developmental competence, as reflected by enhanced blastocyst quality and hatching rate, particularly under oxidative stress conditions. However, since the present evaluation was primarily based on morphological outcomes, further studies including molecular and cellular analyses are warranted to confirm the functional mechanisms underlying these effects. These findings provide valuable evidence supporting the potential application of LA to improve bovine *in vitro* production (IVP) systems.In line with our results, LA supplementation at concentrations ranging from 50 to 300 µM did not significantly impact oocyte nuclear maturation or cleavage rates compared to the control group. This contrasts with previous findings in porcine models, where supplementation with antioxidants such as chlorogenic acid (CGA) significantly improved both maturation and fertilization outcomes (Nguyen et al., 2019). The disparity may stem from species-specific differences in oocyte physiology and metabolic requirements or from differing modes of action between fatty acids like LA and polyphenolic antioxidants like CGA.

Although maturation and cleavage rates remained unaffected, LA supplementation significantly enhanced embryo development post-fertilization. Supplementation with 100–200 µM LA resulted in significantly higher blastocyst formation rates, and higher LA concentrations (200–300 µM) improved blastocyst quality as assessed by morphological grading. High-quality blastocysts are crucial for successful IVP, both for research and agricultural applications, as *in vitro* -derived embryos typically exhibit lower developmental competence compared to their *in vivo* counterparts (Rizos et al., 2002; Lonergan and Fair, 2015). Thus, improving *in vitro* embryo quality remains a key priority in reproductive biotechnology.

This study also examined LA’s potential to mitigate developmental impairment under stress conditions. Heat stress (41 °C) and oxidative stress induced by 1 mM hydrogen peroxide (H_2_O_2_) both significantly reduced blastocyst formation rates. These findings are consistent with extensive evidence that elevated temperature and reactive oxygen species (ROS) can impair embryonic development by disrupting cellular homeostasis and promoting apoptosis (Guerin et al., 2001; Hansen, 2009). The use of atmospheric oxygen (≈ 20%) in *in vitro* systems substantially higher than physiological levels in the female reproductive tract (≈ 5%) further contributes to oxidative stress in IVP environments (Harvey et al., 2002). Importantly, in the present study, LA was supplemented only during the *in vitro* maturation (IVM) period, prior to fertilization and subsequent embryo culture. This design allowed us to evaluate whether maternal cytoplasmic conditioning during IVM could confer greater resilience to subsequent environmental stress.

Notably, LA supplementation at 200 µM during IVM significantly improved the blastocyst rate under oxidative stress conditions (31.5 ± 3.2%) compared to the untreated stress group (18.6 ± 2.7%, p < 0.05), suggesting a protective role. In contrast, under heat stress, LA treatment did not result in a statistically significant improvement, and therefore no biological benefit can be concluded. The present results suggest that LA’s beneficial effects are more evident under oxidative stress rather than thermal stress. While potential explanations such as altered intracellular ROS levels or improved membrane stability have been proposed, these possibilities remain speculative and are not supported by direct evidence in the current study. Further studies investigating mitochondrial activity, ROS dynamics, and gene expression profiles will be necessary to validate these hypotheses. Similar protective effects have been observed with other antioxidant compounds such as melatonin (Kang et al., 2009) and Vitamin E (Barcelona et al., 2025), which have been shown to enhance embryo development and reduce ROS-induced damage. These results collectively suggest that LA supplementation, particularly at concentrations between 100 and 200 µM, can enhance the developmental potential of bovine embryos cultured i*n vitro* under normal conditions. Furthermore, LA at 200 µM demonstrates a protective capacity against the negative effects of oxidative stress on subsequent embryonic development to the blastocyst stage. This aligns with the concept that antioxidants can be beneficial additives to *in vitro* culture systems to counteract the detrimental effects of reactive oxygen species (ROS) generated in the higher oxygen environment of standard IVP systems compared to the female reproductive tract (Rocha-Frigoni et al., 2016). For example, chlorogenic acid, a known antioxidant, was shown to improve maturation, fertilization, and blastocyst formation in porcine oocytes and protect them from heat and oxidative stress (Nguyen et al., 2017)

Although LA is a saturated fatty acid rather than a classical antioxidant, it may influence oxidative stress responses indirectly. Any proposed mechanisms such as changes in mitochondrial function, lipid metabolism, or membrane properties should be regarded as hypothetical, since they were not assessed in this study. Further molecular analyses, including measurements of intracellular ROS, antioxidant enzyme activity (e.g., SOD, GPx), and mitochondrial membrane potential, are required to determine whether LA plays any regulatory role in oxidative pathways. Potential mechanisms include modulation of mitochondrial function, enhancement of lipid metabolism, or alteration of membrane composition that enhances resilience to ROS. These interpretations should be considered speculative and further studies are required to confirm whether LA exerts any direct regulatory effects on oxidative pathways.

Notably, hatching rates did not differ significantly among the treatment groups, suggesting that although LA enhances blastocyst formation and morphological quality, it may have limited impact on subsequent developmental stages under the conditions tested. Hatching is a complex process influenced by multiple factors, including zona pellucida hardening and embryonic metabolic activity, and may require additional or prolonged interventions to elicit measurable improvements. Furthermore, selective hatching of embryos has been proposed as a strategy to enhance implantation rates in humans and other mammals (Cohen et al., 1992; Soom et al., 2003).

## Conclusion

In conclusion, this study demonstrates that Lauric Acid supplementation during bovine IVM enhances embryo developmental competence and provides protection against oxidative stress-induced impairment. These findings support the potential use of LA as a bioactive supplement in IVP systems, particularly for improving embryo yield and quality under suboptimal conditions. Further research is necessary to elucidate the precise mechanisms by which LA confers these effects and to evaluate its integration into standardized IVP protocols.

## Data Availability

Research data is only available upon request.
